# Pinpointing Novel Plasma and Brain Proteins for Common Ocular Diseases: A Comprehensive Cross-Omics Integration Analysis

**DOI:** 10.3390/ijms251910236

**Published:** 2024-09-24

**Authors:** Qinyou Mo, Xinyu Liu, Weiming Gong, Yunzhuang Wang, Zhongshang Yuan, Xiubin Sun, Shukang Wang

**Affiliations:** 1 Department of Biostatistics, School of Public Health, Cheeloo College of Medicine, Shandong University, 44, Wenhuaxi Road, Jinan 250012, China; moqinyou@mail.sdu.edu.cn (Q.M.); liuxinyu202236458@mail.sdu.edu.cn (X.L.); gongweiming@mail.sdu.edu.cn (W.G.); wyz202236493@mail.sdu.edu.cn (Y.W.); yuanzhongshang@sdu.edu.cn (Z.Y.); 2 Institute for Medical Dataology, Shandong University, 12550, Erhuan East Road, Jinan 250003, China

**Keywords:** ocular diseases, plasma proteins, brain proteins, proteome-wide association study, mendelian randomization, colocalization

## Abstract

The pathogenesis of ocular diseases (ODs) remains unclear, although genome-wide association studies (GWAS) have identified numerous associated genetic risk loci. We integrated protein quantitative trait loci (pQTL) datasets and five large-scale GWAS summary statistics of ODs under a cutting-edge systematic analytic framework. Proteome-wide association studies (PWAS) identified plasma and brain proteins associated with ODs, and 11 plasma proteins were identified by Mendelian randomization (MR) and colocalization (COLOC) analyses as being potentially causally associated with ODs. Five of these proteins (protein-coding genes *ECI1*, *LCT*, and *NPTXR* for glaucoma, *WARS1* for age-related macular degeneration (AMD), and *SIGLEC14* for diabetic retinopathy (DR)) are newly reported. Twenty brain-protein–OD pairs were identified by COLOC analysis. Eight pairs (protein-coding genes *TOM1L2*, *MXRA7*, *RHPN2*, and *HINT1* for senile cataract, *WARS1* and *TDRD7* for AMD, *STAT6* for myopia, and *TPPP3* for DR) are newly reported in this study. Phenotype-disease mapping analysis revealed 10 genes related to the eye/vision phenotype or ODs. Combined with a drug exploration analysis, we found that the drugs related to *C3* and *TXN* have been used for the treatment of ODs, and another eight genes (*GSTM3* for senile cataract, *IGFBP7* and *CFHR1* for AMD, *PTPMT1* for glaucoma, *EFEMP1* and *ACP1* for myopia, *SIRPG* and *CTSH* for DR) are promising targets for pharmacological interventions. Our study highlights the role played by proteins in ODs, in which brain proteins were taken into account due to the deepening of eye–brain connection studies. The potential pathogenic proteins finally identified provide a more reliable reference range for subsequent medical studies.

## 1. Introduction

The global prevalence of blindness and vision impairment among middle-aged and elderly individuals has emerged as a public health concern [1], which is largely attributed to diverse ocular diseases (ODs), including cataracts, myopia, glaucoma, age-related macular degeneration (AMD), and diabetic retinopathy (DR). Previous studies have shown that genetic factors play a pivotal role in the development of ODs [2,3]. Recently, genome-wide association studies (GWAS) have identified numerous variants associated with ODs, enhancing our understanding of the genetic basis and heritability of these diseases [4,5,6]. Despite these advancements, the underlying mechanism of ODs remains unclear, preventing the development of effective treatment drugs. It is crucial to investigate in depth how the GWAS risk variants contribute to the onset of ODs and to facilitate the clinical transformations of the genetic findings.

Plasma proteins, originating from diverse organs and tissues, may serve as significant biomarkers in ODs [7,8,9]. Existing studies have illustrated that proteins involved in the development of various ODs are more likely from plasma, providing implications for ocular physiology [10]. In clinical practice, whole blood, as well as its derivatives, are used for the treatment of many ODs [11]. Indeed, plasma proteins are often utilized as effective therapeutic targets, offering valuable insights for drug development across a range of diseases [12]. Moreover, certain brain proteins have been identified not only to impact the central nervous system but also to affect the survival of retinal ganglion cells (RGCs) [13,14,15], the impairment of which could characterize diverse ocular pathologies [16,17]. Studies have unveiled the accumulation of amyloid β in the retina of AMD patients, akin to its presence in the brains of Alzheimer’s disease (AD) patients and glaucoma patients [16]. Indeed, brain proteins are emerging as novel therapeutic targets for ODs [18].

Integrating genetics and proteomics can help open the black box from the genotypes to ODs and identify potential protein–OD associations, thus benefiting proteomics-driven drug target discovery and clinical transformations of GWAS findings. Fortunately, there are many publicly available GWASs and protein quantitative trait loci (pQTL) summary statistics, parallelized with several well-developed statistical genetics methods, to support such an integration analysis. In particular, the proteome-wide association study (PWAS) analysis aims to identify the associations between proteins and diseases by integrating GWAS and pQTL studies. PWAS has been extensively applied to neuropsychiatric, cardiovascular, and cerebrovascular diseases [19,20,21], providing a deeper biological understanding of the pathogenesis and subsequently fostering the identification of potential drug targets. Meanwhile, mendelian randomization (MR) [22] has become a common statistical tool to investigate the causal effect of an exposure (protein) on the outcome trait (OD) by leveraging genetic variants. In addition, Bayesian colocalization (COLOC) analysis can be utilized to examine and identify the shared causal variants between proteins and ODs. Different analysis techniques typically require distinct model assumptions; the findings from only a single method may be less robust and convincing. The integration of different analysis methods can systematically investigate the relationship between proteins and ODs.

**Figure 1:**
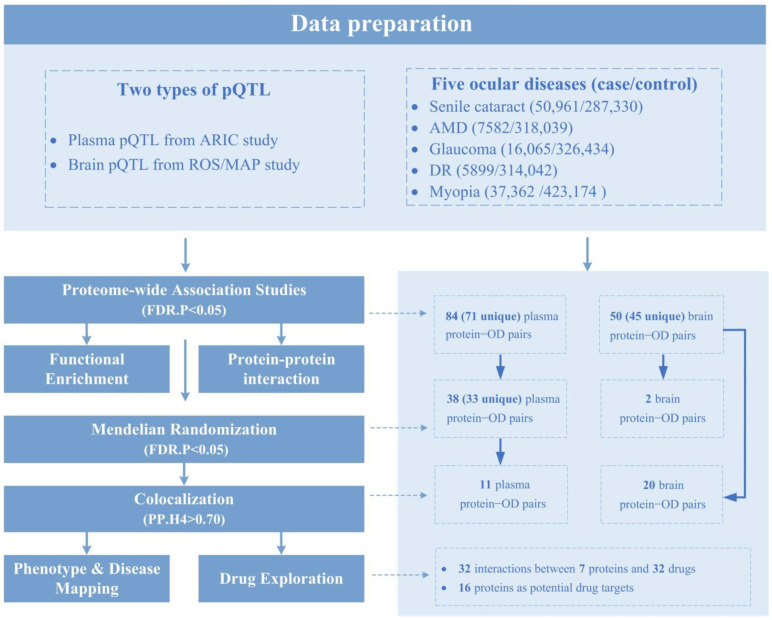
Flowchart. We through a cutting-edge systematic analytic framework, aimed to identify the proteins associated with five common ocular diseases, including senile cataract, glaucoma, AMD, myopia, and DR. The left side shows the specific methods used and the right side corresponds to the results of each method. Abbreviations: ARIC, Atherosclerosis Risk In Communities; AMD, Age-related macular degeneration; DR, Diabetic retinopathy; FDR, false discovery rate; OD, Ocular disease; pQTL, protein quantitative trait loci; ROS/MAP, Religious Orders Study/Memory and Aging Project.

Here, we, through a cutting-edge systematic analytic framework (Figure 1), aimed to identify the proteins associated with five common ODs, including senile cataract, glaucoma, AMD, myopia, and DR. Specifically, we first integrated large-scale GWAS data and pQTL data (including both plasma and brain proteins) for PWAS analysis, enabling the initial identification of protein-coding genes linked to ODs. We also performed a functional enrichment analysis to reveal the underlying biological processes and pathways. Then, we performed an MR analysis to identify potential protein–OD causal pairs, followed by COLOC analysis to pinpoint shared genetic variations between proteins and ODs. We also performed phenotype and disease mapping analysis on the Mouse Genome Informatics (MGI) platform. Finally, the drug–gene interaction database (DGIdb) was utilized to prioritize potential drug targets.

## 2. Results

### 2.1. PWAS Identified 134 Protein–OD Pairs

**Figure 2:**
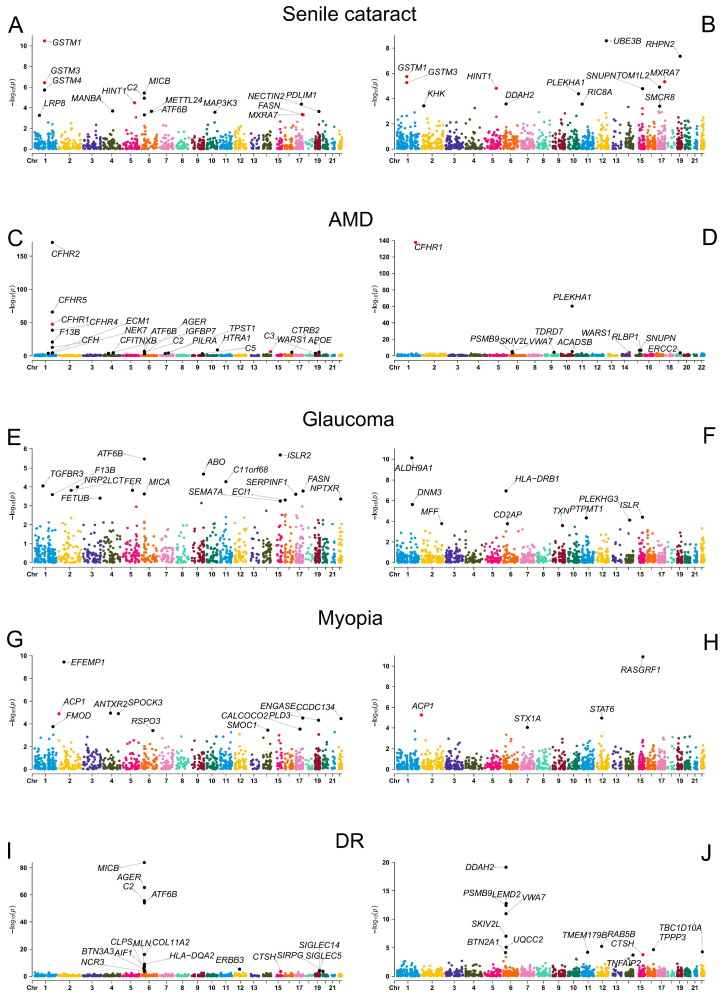
Manhattan plots of PWAS analysis for 5 ODs. (**A**) Plasma-protein–Senile cataract associations. (**B**) Brain-protein–Senile cataract associations. (**C**) Plasma-protein–AMD associations. (**D**) Brain-protein–AMD associations. (**E**) Plasma-protein–Glaucoma associations. (**F**) Brain-protein–Glaucoma associations. (**G**) Plasma-protein–Myopia associations. (**H**) Brain-protein–Myopia associations. (**I**) Plasma-protein–DR associations. (**J**) Brain-protein–DR associations. Each dot on the x-axis represents a gene, and the association strength on the y-axis represents the −log10 (*p*) of PWAS. Proteome-wide significance level was set at 0.05 which adjusted by false discovery rate (FDR). Genes that were proteome-wide significant in both plasma and brain proteomes are shown in red. Abbreviations: AMD, Age-related macular degeneration; DR, Diabetic retinopathy.

We conducted A PWAS analysis by integrating GWASs of five ODs with both plasma-protein pQTL data and brain-protein pQTL data, respectively. A total of 84 plasma-protein–OD pairs (71 unique protein-coding genes) were identified, including 15 for senile cataract, 25 for AMD, 16 for glaucoma, 12 for myopia, and 16 for DR (Figure 2, Table S2). In addition, a total of 50 brain-protein–OD pairs (45 unique protein-coding genes) were identified, including 13 for senile cataract, 11 for AMD, 9 for glaucoma, 4 for myopia, and 13 for DR (Figure 2, Table S3). Two proteins were identified in no less than three ODs, namely, plasma-protein-coding gene *ATF6B* was identified in senile cataract (FDR.*p* = 4.66 × 10^−2^), AMD (FDR.*p* = 6.80 × 10^−3^), glaucoma (FDR.*p* = 2.26 × 10^−3^), and DR (FDR.*p* = 3.10 × 10^−52^); plasma-protein-coding gene *C2* was identified in senile cataract (FDR.*p* = 3.18 × 10^−3^), AMD (FDR.*p* = 5.42 × 10^−4^), and DR (FDR.*p* = 9.97 × 10^−54^). In addition to this, nine other proteins (four plasma proteins and five brain proteins) were also found in both ODs. For example, plasma-protein-coding gene *AGER* was identified in AMD (FDR.*p* = 4.79 × 10^−2^) and DR (FDR.*p* = 2.32 × 10^−63^), brain-protein-coding gene *DDAH2* was identified in senile cataract (FDR.*p* = 4.02 × 10^−2^) and DR (FDR.*p* = 1.16 × 10^−16^), and brain-protein-coding gene *PLEKHA1* was identified in senile cataract (FDR.*p* = 7.45 × 10^−3^) and AMD (FDR.*p* = 4.40 × 10^−58^). The fact that certain proteins were involved in multiple ODs may suggest that there are some developmental commonalities between ODs.

In addition, eight protein-coding genes were identified in both plasma and brain PWAS analysis, namely *GSTM1* (plasma FDR.*p* = 4.42 × 10^−8^, brain FDR.*p* = 9.85 × 10^−4^), *GSTM3* (plasma FDR.*p* = 2.48 × 10^−4^, brain FDR.*p* = 1.75 × 10^−3^), *HINT1* (plasma FDR.*p* = 7.15 × 10^−3^, brain FDR.*p* = 3.27 × 10^−3^), *MXRA7* (plasma FDR.*p* = 4.66 × 10^−2^, brain FDR.*p* = 1.75 × 10^−3^) for senile cataract, *CFHR1* (plasma FDR.*p* = 1.39 × 10^−45^, brain FDR.*p* = 3.38 × 10^−135^) and *WARS1* (plasma FDR.*p* = 6.22 × 10^−5^, brain FDR.*p* = 1.80 × 10^−2^) for AMD, *ACP1* (plasma FDR.*p* = 4.30 × 10^−3^, brain FDR.*p* = 4.46 × 10^−3^) for myopia, and *CTSH* (plasma FDR.*p* = 2.46 × 10^−2^, brain FDR.*p* = 2.31 × 10^−2^) for DR. Specifically, only the proteins encoded by *ACP1* (plasma PWAS.*Z* = −4.36, brain PWAS.*Z* = 4.55) and *WARS1* (plasma PWAS.*Z* = −3.53, brain PWAS.*Z* = 3.89) showed discordant associations.

**Figure 3:**
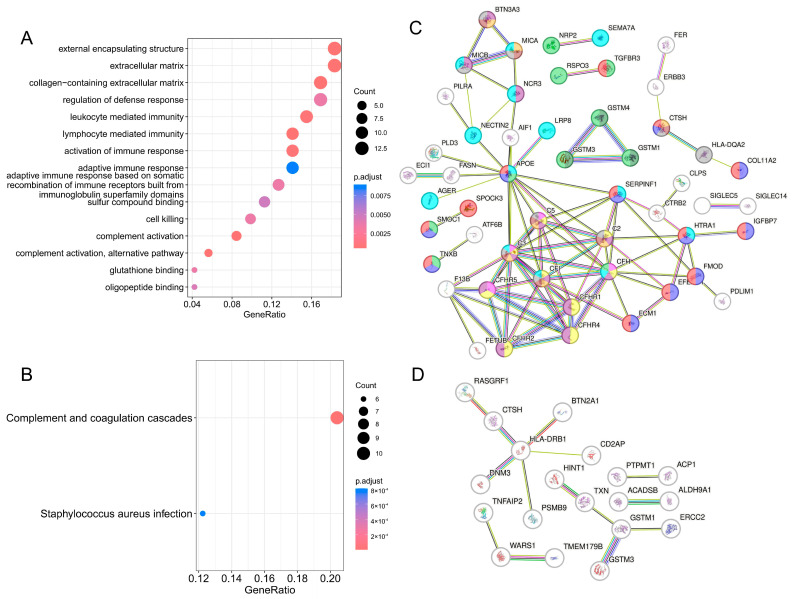
Bubble plots for enrichment analysis and PPI network. (**A**) Bubble plots for GO enrichment analysis of plasma proteins. (**B**) Bubble plots for KEGG enrichment analysis of plasma proteins. The size of the dots indicates the number of target genes; different colors of the dots indicate different ranges of *p*-value. (**C**) PPI network of plasma proteins. (**D**) PPI network of brain proteins. Red line indicates the presence of fusion evidence. Green line indicates neighborhood evidence. Blue line indicates cooccurrence evidence. Purple line indicates experimental evidence. Yellow line indicates text-mining evidence. Light blue line indicates database evidence. Black line indicates co-expression evidence. Proteins labeled with Salmon pink indicate that they are enriched into the GO-term “extracellular matrix”. Proteins labeled with Vista blue indicate that they are enriched into the GO-term “collagen-containing extracellular matrix”. Proteins labeled with Celeste indicate that they are enriched into the GO-term “regulation of defense response”. Proteins labeled with Sunset indicate that they are enriched into the GO-term “lymphocyte-mediated immunity”. Proteins labeled with Lilac indicate they are enriched into the GO-term “activation of immune response”. Proteins labeled with Silver indicate that they are enriched into the GO-term “adaptive immune response”. Proteins labeled with Rosy brown indicate that they are enriched into the GO-term “adaptive immune response based on somatic recombination of immune receptors built from immunoglobulin superfamily domains”. Proteins labeled with Celadon indicate that they are enriched into the GO-term “sulfur compound binding”. Proteins labeled with Cream indicate that they are enriched into the GO-term “complement activation”. Proteins labeled with Violet indicate that they are enriched into the GO-term “complement activation, alternative pathway”. Proteins labeled with Cambridge blue indicate that they are enriched into the GO-term “glutathione binding”. Proteins labeled with White indicate that they are not enriched into GO terms.

We further performed an enrichment analysis of these proteins and mapped the protein–protein interaction (PPI) network. For plasma proteins, we identified 45 significant Gene Ontology (GO) terms (Figure 3, Table S14), such as the activation of an immune response (FDR.*p* = 7.96 × 10^−4^), the regulation of a defense response (FDR.*p* = 3.53 × 10^−3^), glutathione-binding (FDR.*p* = 3.53 × 10^−3^), and two significant Kyoto Encyclopedia of Genes and Genomes (KEGG) terms (Figure 3, Table S15), including complement and coagulation cascades (FDR.*p* = 3.30 × 10^−9^) and a staphylococcus aureus infection (FDR.*p* = 8.04 × 10^−4^). Figure 3A shows only the top 15 Go terms, and Figure 3C details the interactions between the significant plasma proteins identified by PWAS. Although the brain proteins identified by PWAS were not enriched for any pathway (Tables S16 and S17), the interaction enrichment indicated that these proteins were at least partially biologically connected as a group (Figure 3).

### 2.2. MR Identified 40 Protein–OD Pairs

**Figure 4:**
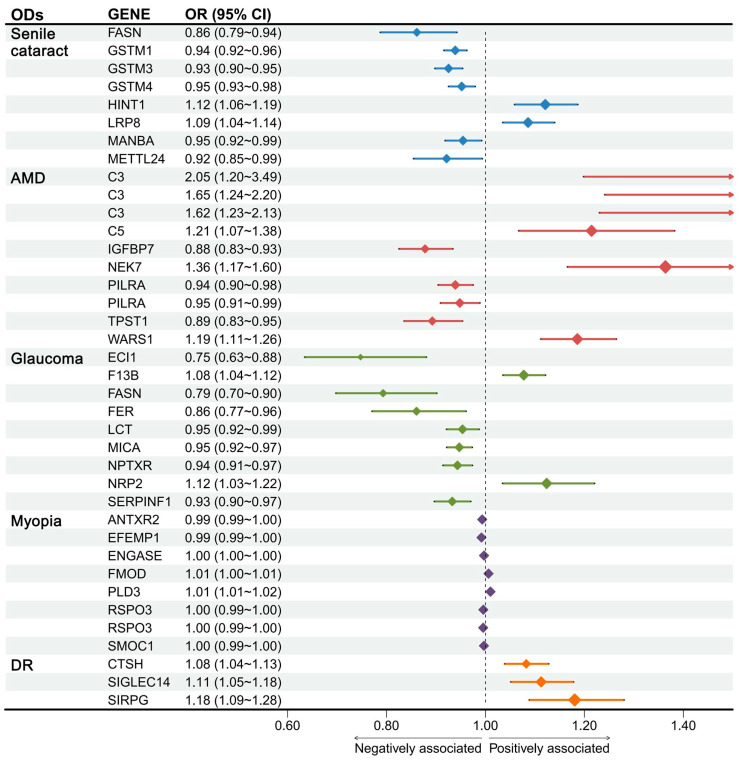
Forest plot of MR results for plasma-protein–OD pairs that have been tested for heterogeneity and pleiotropy. Abbreviations: ODs, Ocular diseases; AMD, Age-related macular degeneration; DR, Diabetic retinopathy; OR, Odds ratio; CI, Confidence Interval. Since the concentration of plasma-protein-coding genes *C3*, *PILRA,* and *RSPO3* were measured by multiple aptamers (SOMAmer) corresponding to multiple SeqIDs, several different forest plots/ORs were obtained.

For protein–OD pairs identified by PWAS, 40 potential casual protein–OD pairs were further confirmed by MR, including 38 plasma-protein–OD pairs (8 for senile cataract, 10 for AMD, 9 for glaucoma, 8 for myopia, and 3 for DR). In addition, all instrumental variables (IVs) extracted from each protein had *F*-statistics greater than 10, ruling out the issue of weak IVs, and the MR–Steiger test showed no reverse causality. For proteins with more than three IVs, we examined heterogeneity using the MR–Heterogeneity method and horizontal pleiotropy using intercepts from the MR–Egger method and assessed the robustness of the MR estimates by a leave-one-out analysis. All results illustrated the nonexistence of heterogeneity and pleiotropy. Finally, these 38 plasma-protein–OD pairs remained (Figure 4, Table S4). In addition, approximately 60% (23/38) of the protein–OD pairs showed negative causality, indicating that these proteins may play a protective role in the corresponding OD, such as protein-coding genes *GSTM3* for senile cataract (odds ratio (OR) = 0.926, 95% confidence interval (CI) (0.898, 0.954), FDR.*p* = 3.59 × 10^−6^), *IGFBP7* for AMD (OR = 0.878, 95%CI (0.825, 0.934), FDR.*p* = 3.12 × 10^−4^), *EFEMP1* (OR = 0.992, 95%CI (0.988, 0.996), FDR.*p* = 2.51 × 10^−4^) for myopia, and *SIRPG* (OR = 1.181, 95%CI (1.089, 1.280), FDR.*p* = 4.81 × 10^−4^) for DR. Results of other MR methods and sensitivity analyses are shown in Tables S8–S11.

Most of the proteins identified by brain PWAS analysis only have one single IV; thus, MR analysis mainly relies on the Wald ratio method. After correction for multiple testing, we identified two potential causal genes (Table S5), namely *KHK* associated with senile cataract (OR = 1.089, 95%CI (1.035–1.145), FDR.*p* = 1.44 × 10^−3^) and *CFHR1* (OR = 3.049, 95%CI (2.636–3.527), FDR.*p* = 7.04 × 10^−50^). The IVs extracted from each protein had F-statistics greater than 10, and the MR Steiger test showed no reverse causality. The results of other MR methods and sensitivity analyses are shown in Tables S12 and S13.

### 2.3. COLOC Validated Causal Relationships of 11 Plasma-Protein–OD Pairs

To rule out the bias from MR analysis due to the proteins and ODs being driven by distinct variants within linkage disequilibrium (LD), we performed a COLOC analysis on plasma-protein–OD pairs identified from the MR analysis. A total of 11 unique protein-coding genes posterior probability (PP) of H4 > 0.70 finally remained, including *GSTM3* for senile cataract, *WARS1*, *C3*, *IGFBP7*, and *PILRA* for AMD, *ECI1*, *LCT*, and *NPTXR* for glaucoma, *EFEMP1* for myopia, and *SIRPG* and *SIGLEC14* for DR. The PP.H4 of these 11 proteins, as well as their coding genes, are provided (Table 1); six of them have been confirmed in previous studies, suggesting that these genes may affect the corresponding ODs through the encoded proteins, and five novel genes were reported in this study. Detailed results are shown in Table S6.

### 2.4. COLOC Validated 20 Out of 50 Brain-Protein–OD Pairs Identified by PWAS

We observed that the F-statistics of most IVs for the 50 brain proteins identified from PWAS were too small (F-statistics < 10) to meet the strong correlation criteria between IVs and exposure, and a weak instrumental bias was more likely to be produced [23]. In this case, the results from the MR analysis may be less reliable. Here, we chose to directly perform a COLOC analysis for 50 brain-protein–OD pairs from the PWAS analysis. Ultimately, 20 unique brain proteins (Table 1) were identified (PP.H4 > 0.70), including 5 for senile cataract, 4 for AMD, 5 for glaucoma, 3 for myopia, and 3 for DR. A total of 8 out of these 20 proteins are newly reported in our study. Detailed results are shown in Table S7.

### 2.5. Phenotype-Disease Mapping Analysis for COLOC-Associated Proteins

For the 31 COLOC-associated proteins (11 plasma proteins and 20 brain proteins), we further performed the phenotype-disease mapping analysis to examine whether these proteins were related to vision/eye phenotype or ODs. Eventually, we found four plasma-protein-coding genes (*WARS1*, *C3*, *LCT*, and *EFEMP1*) and six brain-protein-coding genes (*TOM1L2*, *CFHR1*, *WARS1*, *RLBP1*, *TDRD7*, and *CTSH*) were associated with eye/vision phenotype or ODs (Table 1). Specifically, *CFHR1* was associated with AMD, *WARS1* was associated with abnormal lens morphology and cataracts, *LCT* was associated with abnormal retinal morphology and decreased total retina thickness, and *CTSH* was associated with abnormal retina outer nuclear layer morphology, decreased total retina thickness, and abnormal retina inner nuclear layer morphology.

### 2.6. Candidate Druggable Targets

As most drugs exert their therapeutic effects through targeting proteins, we finally explored if the 11 plasma proteins and 20 brain proteins identified through the comprehensive analysis can serve as potential therapeutic targets. We searched the drug–gene interactions from DGIdb and identified four interactions (Table S18) between two plasma-protein-coding genes (*GSTM3*, *C3*) and four drugs and 28 interactions (Table S19) between five brain-protein-coding genes (*GSTM3*, *HINT1*, *TXN*, *STAT6*, and *ACP1*) and 28 drugs. Through druggability explorations, a total of eight plasma-protein-coding genes (*GSTM3*, *WARS1*, *C3*, *IGFBP7*, *ECI1*, *LCT*, *EFEMP1*, and *SIRPG*) (Table S20) and eight brain-protein-coding genes (*GSTM3*, *CFHR1*, *WARS1*, *PTPMT1*, *TXN*, *STAT6*, *ACP1*, and *CTSH*) (Table S21) were identified to be the potential targets for drug therapy intervention. These findings are expected to promote and facilitate the development of specific drugs for ODs.

## 3. Discussion

In this study, we performed a comprehensive omics-integration analysis, including PWAS, MR, and COLOC methods, to identify the plasma proteins and brain proteins that are causally associated with five common ODs. A total of 134 protein–OD pairs were identified by PWAS, and functional enrichment analysis demonstrated that OD-associated plasma proteins significantly enriched immune-related biological processes and pathways, such as complement activation, lymphocyte-mediated immunity, and leukocyte-mediated immunity. Further MR and COLOC analyses identified 11 plasma-protein–OD pairs. Considering the weak instrumental variable bias of brain-protein–OD pairs in the MR analysis, we directly performed a COLOC analysis for 50 brain-protein–OD pairs from the PWAS analysis, and 20 pairs were identified. Among the 11 plasma-protein–OD pairs, 6 pairs (protein-coding genes *GSTM3* for senile cataract, *C3*, *IGFBP7* and *PILRA* for AMD, *EFEMP1* for myopia, *SIRPG* for DR) have been identified in previous studies, while the other 5 pairs (protein-coding genes *ECI1*, *LCT* and *NPTXR* for glaucoma, *WARS1* for AMD, *SIGLEC14* for DR) are newly reported. Among the 20 brain-protein–OD pairs, 12 associations have been identified in previous studies, while the other 8 associations (related to genes *TOM1L2*, *WARS1*, *MXRA7*, *RHPN2*, *HINT1*, *TDRD7*, *STAT6*, and *TPPP3*) are newly reported in this study. In addition, we characterized 10 of them as related to vision/eye phenotypes or ODs. These proteins would not only benefit the pathogenesis of ODs but could also serve as novel therapeutic targets. Combined with a drug exploration analysis, we classified these proteins into three categories: the proteins that have been reported and have relevant drugs used to treat ODs, the proteins that have been reported and show potential for the development of drugs, and the proteins that are newly reported in this study.

The drugs related to *C3* and *TXN* have been used for the treatment of ODs. AMD is a prevalent OD driven by a complement, and *C3* is the central complement component and a key inflammatory protein activated in AMD [24]; the drugs related to *C3*, such as compstatin and pegcetacoplan, are used for the treatment of AMD [25,26]. Glaucoma is characterized by the progressive loss of RGCs. Several studies have confirmed that the overexpression of *TXN* increases RGC’s survival in treating glaucoma [27]. Moreover, *TXN* can improve the antioxidant capacity of the retina [28], and it has been experimentally demonstrated that the drug Gambogic acid, related to *TXN*, can be used to treat DR by inhibiting retinal angiogenesis [29].

Some proteins reported in this study may provide the potential for drug development for the corresponding ODs. For senile cataracts, *GSTM3*, which is both identified in plasma and brain proteins and the downregulation of which in the lens epithelial cells (LECs) made LECs more susceptible to oxidative stress, induces the formation of cataracts [30]. For AMD, *IGFBP7* is an angiogenesis inhibitor and is involved in the development of neovascular AMD [31]. *CFHR1* plays an important role in complement regulation and is considered to be protective against AMD [32]. For glaucoma, *PTPMT1* is associated with optic nerve atrophy [33] and elevated intraocular pressure [34,35], which are features of glaucoma and increase the risk of glaucoma. For myopia, *EFEMP1* is highly expressed in the corneal and choroidal retinal pigment epithelium [36] and is a potential biomarker of choroid thickness changes in myopia [37]. *ACP1* is significantly upregulated in retinal protein expression in myopic guinea pigs, and a joint meta-analysis has identified *ACP1* as a novel locus associated with myopia in European pedigrees [38]. The phosphorylation function of the *ACP1* (Acid Phosphatase 1) protein is important in myopic eye growth [39]. Regarding the inconsistent association of *ACP1* in plasma and brains, the PWAS analysis may suggest tissue specificity. For DR, a proteome-wide MR study [40] highlights *SIRPG* as a significant marker for it. *CTSH* polymorphisms are associated with a reduced risk of progression to proliferative diabetic retinopathy [41]. Interestingly, several studies [42,43,44] suggested a potential link between elevated *SIRPG* and *CTSH* levels and diabetes susceptibility.

The 13 newly reported genes are more likely to be associated with eye-related diseases, which can be supported by phenotype-disease mapping analysis, animal experiments, and GWAS studies. In particular, the mapping analysis showed that *WARS1*, *LCT*, *TDRD7*, and *TOM1L2* were associated with eye/visual phenotypes. Among them, gene *WARS1,* both identified in plasma and brain, encodes protein tryptophan-TRNA synthetase, which has vasopressor activity [45] and can be used as a potential anti-angiogenic agent for the treatment of neovascular ODs [46]. Again, similar to *ACP1*, tissue specificity may need attention. The protein encoded by *TDRD7* is associated with lens development and is thought to be linked to cataracts, microphthalmia, and Peters abnormalities [47]. Animal experiments have also shown that the protein encoded by *STAT6* plays a key role in the formation of retinal structures [48] and mediates ocular atopic dermatitis in mice [49]. In GWAS studies, it was found that *RHPN2* and *MXRA7* are associated with macular thickness [50] and conical cornea [51], respectively. Thus, it could be speculated that the newly reported genes can provide the potential for the development of targeted drugs for corresponding ODs. Our study is not without limitations. First, we only focused on European ancestry due to the large-scale pQTL data, and GWASs of five ODs were only available in the European population; thus, the findings cannot be directly extended to other populations. Second, we are unable to validate the 20 brain-protein–OD pairs using an MR analysis due to the weak instrument issue for the IVs of the brain proteins, which may be due to the small sample size of brain pQTL data. Finally, our findings need to be further validated by experimental studies or clinical trials.

## 4. Materials and Methods

### 4.1. Data Sources

#### 4.1.1. GWAS Summary Data

The GWAS summary statistics for myopia were obtained from the UK Biobank (UKB) cohort, which included 37,362 cases and 423,174 controls. The GWASs of the remaining four ODs were obtained from the FinnGen [52], including senile cataract (50,961 cases and 287,330 controls), AMD (7582 cases and 318,039 controls), glaucoma (16,065 cases and 326,434 controls), and DR (5899 cases and 314,042 controls). The above GWAS data are all of European descent and have the highest number of cases of the available databases to ensure statistical power. All GWASs were ethically approved, with additional information available in Table S1.

#### 4.1.2. Human Plasma pQTL Data

The associations between genetic variants and protein expression levels, known as pQTL data, can be obtained through pQTL analysis and are commonly utilized to integrate with GWAS data to identify the proteins related to complex diseases. The pQTL data were obtained from the European Ancestry subcohort of the Atherosclerosis Risk in Communities (ARIC) study [53], which involved 4657 plasma proteins from 7213 individuals. The plasma proteins were measured using an aptamer (SOMAmer)-based approach on the V4 platform by SomaLogic Inc. (Sales and high-throughput, CLIA-certified lab 2945 Wilderness Place, Boulder, CO, USA). Association analyses began with linear regression adjustments based on covariates, followed by linking rank-inverse normalized residuals to genetic variance. The detailed quality control and analysis procedure were provided in the original publications. The 1348 significant cis-heritable plasma proteins (i.e., the nonzero cis-heritability with *p*-value < 0.01) with available imputation weights were used for PWAS analysis.

#### 4.1.3. Human Brain pQTL Data

The human brain pQTL was obtained from the dorsolateral prefrontal cortex of post-mortem brain samples donated by 376 participants of European Descent from the Religious Orders Study/Memory and Aging Project (ROS/MAP) [19]. Proteome sequencing was conducted using isobaric tandem mass tag peptide labeling and analyzed via liquid chromatography-mass spectrometry. After quality control, 8356 proteins remained in the pQTL analysis. Genotypes were obtained from whole genome sequencing or genome-wide genotyping. A total of 1475 proteins showing significant associations with genetic variation were used in the PWAS analysis.

### 4.2. Statistical Analysis

#### 4.2.1. Proteome-Wide Association Studies

PWAS has become an efficient tool for exploring protein-disease associations by integrating the genetic imputation modeling of protein expression with GWAS. In this study, we used the FUSION pipeline, incorporating imputation weights for plasma proteins from the ARIC study and brain proteins from the ROS/MAP datasets. FUSION employs input single nucleotide polymorphisms (SNP)-protein imputation weights to predict the abundance of each protein in the GWAS for each OD, followed by association analysis between the predicted protein abundance and ODs [54]. The European 1000 Genomes panel served as the reference for LD data, and FUSION was implemented with default settings. Proteins with a *p*-value < 0.05 adjusted by FDR were declared to be significant, which have been commonly used in the previous literature [55,56,57].

#### 4.2.2. Functional Enrichment Analysis and Protein–Protein Interaction Network

To explore the underlying biological mechanisms associated with OD-related proteins identified by PWAS, we conducted the functional enrichment analysis using the “clusterProfiler” package (https://github.com/YuLab-SMU/clusterProfiler) (accessed on 8 September 2023) in R 4.2.2. by utilizing gene sets derived from two databases—GO and KEGG pathways. A *p*-value < 0.05 corrected by FDR was selected for functional annotation clustering. Additionally, we used STRING (https://string-db.org/) (accessed on 8 September 2023) to create PPI networks, allowing us to examine the connections among important proteins identified by PWAS. The STRING database encompasses a phylogenetically diverse collection of 12,535 high-quality genomes [58].

#### 4.2.3. Mendelian Randomization Analysis

We performed a two-sample MR analysis, paralleled with a set of sensitivity analyses, on the protein–OD pairs identified by the PWAS analysis. The MR analysis conforms to the STROBE-MR Statement [59], mainly involving instrumental variable selection, instrumental variable assessment, primary MR analysis, and sensitivity analysis. We first selected IVs for the proteins from their cis-pQTLs. We performed LD clumping, with the threshold of r^2^ < 0.01 in the 1000 kb cis-region, to select the independent instrumental SNPs. LD calculation was based on the European LD reference panel in the 1000 Genomes. Meanwhile, due to the different number of cis-SNPs for different proteins in the cis-regions, we adopted a protein-specific Bonferroni-corrected *p*-value threshold (0.05/the number of SNPs in the cis-region of each protein) to declare significant pQTLs. After harmonizing the effect alleles of IVs in pQTLs data and in outcome GWAS data of each OD, the retained SNPs were used for MR analysis. In addition, we performed the MR Steiger directionality test to alleviate the issue of reverse causality. We also used the *F*-statistics to evaluate the issue of weak instrumental bias and removed those IVs with *F*-statistics less than 10 [60,61].

We used the Wald ratio test for those proteins with only one IV and the inverse variance weighted (IVW) method for those proteins with more than one IV [62]. Both MR–Egger and weighted median methods were also used to evaluate the consistency of the findings across different MR methods [63,64]. We used OR to characterize the causal effect of proteins on each OD, quantifying the change in OD risk per one SD change in protein abundance. In addition, for proteins with more than three IVs, we further assessed heterogeneity using Cochran’s Q-value and tested for horizontal pleiotropy using the MR–Egger intercept.

Finally, to assess the robustness of the MR results, we also applied the leave-one-out method to test whether the removal of one IV could change the overall IVW estimate. In cases where any outliers were identified, we removed them and re-conducted the MR causal analysis. The *p*-value was corrected using the FDR method, and protein–OD pairs with FDR.*p* < 0.05 were considered to have potential causal associations. All the above analyses were performed using the “TwoSampleMR” package (https://github.com/MRCIEU/TwoSampleMR) (accessed on 10 September 2023) in R 4.2.2.

#### 4.2.4. Bayesian Colocalization Analysis

We performed COLOC analysis to assess whether the proteins and ODs shared common genetic variants. Of note is that COLOC analysis can help evaluate the bias in MR analysis due to LD correlation. We adopted the commonly used Bayesian model-coloc, which uses an approximate Bayesian factor to calculate the PP of all possible configurations between proteins and ODs: (1) H0: no association with either proteins or ODs; (2) H1: association with proteins, not with ODs; (3) H2: associated with ODs, not with proteins; (4) H3: associated with both proteins and ODs, two distinct SNPs; (5) H4: associated with both proteins and ODs, one shared SNP [65]. The PP of each configuration is represented by PP0, PP1, PP2, PP3, and PP4. We mainly focused on H4 and considered strong evidence of COLOC when PP.H4 is larger than 0.70 [20]. These PPs were calculated using the Bayesian “coloc. abf” function and “coloc” package in R (http://cran.r-project.org/web/packages/coloc) (accessed on 12 September 2023).

#### 4.2.5. Phenotype and Disease Mapping Analysis

To further validate our results, we performed phenotype-disease mapping analysis on all proteins identified by COLOC using the Human-Mouse Disease Correlation (HMDC) tool (https://www.informatics.jax.org/humanDisease.shtml) (accessed on 15 September 2023) from the MGI homepage, a tool for exploring mouse and human phenotype-disease relationships designed to highlight known experimental mouse disease models. In our study, we focused on vision/eye phenotype and ODs.

#### 4.2.6. Druggable Targets Exploration

To explore the possibility of targeting the identified proteins with existing drugs or as potential gene targets for drug development, we thoroughly analyzed the interactions between these proteins (or genes) and drugs using DGIdb (version 4.0) (https://www.dgidb.org/) (accessed on 1 October 2023). DGIdb provides advanced search and filtering capabilities for drug–gene interactions and drug genomic information, drawing from over 30 reliable sources such as DrugBank, PharmGKB, ChEMBL, Drug Target Commons, and Therapeutic Target Database, and more, housing 40,000 genes and 10,000 drugs, and encompassing over 100,000 drug–gene interactions or falling within one of 42 potential drug–gene classes. Widely relied upon, DGIdb effectively prioritizes potential drug targets for various diseases [66,67].

## 5. Conclusions

Focusing on the population with European ancestry, we identified the potentially causal brain and plasma proteins for ODs. The potential pathogenic proteins finally identified provide a more reliable reference range for subsequent medical studies.

## Figures and Tables

**Table 1:** A total of 31 protein–OD pairs identified by the systematic analysis.

ODs	Gene	Brain Protein			Gene	Plasma Protein				
		PWAS		COLOC		PWAS		MR		COLOC
		Z	*p*	PP.H4		Z	*p*	OR	*p*	PP.H4
Senile catarct	GSTM3 *^,D^	−4.55	5.38 × 10^−6^	0.977	GSTM3 *^,D^	−5.08	3.74 × 10^−7^	0.93	4.85 × 10^−7^	0.909
	TOM1L2 ^M^	−4.37	1.22 × 10^−5^	0.934	-	-	-	-	-	-
	MXRA7	−4.58	4.65 × 10^−6^	0.982	-	-	-	-	-	-
	RHPN2	−5.47	4.44 × 10^−8^	0.988	-	-	-	-	-	-
	HINT1	4.33	1.52 × 10^−5^	0.964	-	-	-	-	-	-
AMD	WARS1 ^M^	3.89	1.00 × 10^−4^	0.880	WARS1 ^D,M^	−3.53	4.70 × 10^−7^	1.19	2.04 × 10^−7^	0.936
	CFHR1 *^,D,M^	25.04	2.08 × 10^−138^	0.737	C3 *^,D,M^	9.03	1.68 × 10^−19^	2.05	8.71 × 10^−3^	0.999
	RLBP1 *^,M^	5.23	1.74 × 10^−7^	0.920	IGFBP7 *^,D^	−3.70	2.16 × 10^−4^	0.88	3.75 × 10^−5^	0.754
	TDRD7 ^M^	4.01	5.95 × 10^−5^	0.923	PILRA *	−3.99	6.60 × 10^−5^	0.95	1.28 × 10^−2^	0.866
Glaucoma	DNM3 *	−4.72	2.34 × 10^−6^	0.897	ECI1 ^D^	−3.48	4.94 × 10^−4^	0.75	4.93 × 10^−4^	0.711
	PTPMT1 *^,D^	4.07	4.65 × 10^−5^	0.861	LCT ^D,M^	−3.79	1.52 × 10^−4^	0.95	6.97 × 10^−3^	0.899
	ISLR *	−4.11	3.98 × 10^−5^	0.759	NPTXR	−3.52	4.37 × 10^−4^	0.94	3.03 × 10^−4^	0.883
	MFF *	−3.76	1.68 × 10^−4^	0.987	-	-	-	-	-	
	TXN *^,D^	−3.65	2.59 × 10^−4^	0.926	-	-	-	-	-	-
Myopia	STAT6 ^D^	−4.40	1.09 × 10^−5^	0.957	EFEMP1 *^,D,M^	−6.27	3.60 × 10^−10^	0.99	6.28 × 10^−5^	0.993
	RASGRF *	−6.77	1.27 × 10^−11^	0.887	-	-	-	-	-	-
	ACP1 *^,D^	4.55	5.49 × 10^−6^	0.886	-	-	-	-	-	-
DR	RAB5B *	−4.54	5.52 × 10^−6^	0.761	SIRPG *^,D^	3.69	2.20 × 10^−4^	1.18	6.01 × 10^−5^	0.783
	CTSH *^,D,M^	3.76	1.71 × 10^−4^	0.856	SIGLEC14	3.70	2.14 × 10^−4^	1.11	2.39 × 10^−4^	0.985
	TPPP3	−4.26	2.02 × 10^−5^	0.922	-	-	-	-	-	-

Note: We identified candidate genes by COLOC using genes remained by MR for plasma-OD pairs and PWAS for brain–OD pairs. * Genes have been shown to be associated with corresponding ODs in previous studies. ^D^ Genes are all known or potentially druggable genes. ^M^ Genes interact with vision/eye phenotype or ODs. Abbreviations: AMD, Age-related macular degeneration; DR, Diabetic retinopathy; MR, Mendelian randomization; ODs, Ocular diseases; OR, Odds ratio; PWAS, Proteome-Wide Association study; COLOC, Colocilization; PP.H4, Posterior Probability of H4.

## Data Availability

The datasets generated and/or analyzed during the current study are publicly available. GWAS summary data of myopia is available from the MRC IEU OpenGWAS database (GWAS ID: ukb-b-6353) at https://gwas.mrcieu.ac.uk/ (accessed on 1 September 2023), summary statistics for the remaining four ODs from the Finngen database. (https://www.finngen.fi/en/access_results) (accessed on 1 April 2023) Plasma pQTL data can be downloaded at http://nilanjanchatterjeelab.org/pwas/ (accessed on 1 September 2023). Brain pQTL data can be downloaded at https://www.synapse.org/#!Synapse:syn23191787 (accessed on 5 September 2023).

## Supplementary Materials

The following supporting information can be downloaded at https://www.mdpi.com/article/10.3390/ijms251910236/s1.

## Abbreviations

AD: Alzheimer’s disease; AMD, Age-related macular degeneration; ARIC, Atherosclerosis Risk In Communities; COLOC, Colocalization; DGIdb, Drug–Gene Interaction Database; DR, Diabetic Retinopathy; FDR, False Discovery Rate; GWAS, Genome-Wide Association Studies; GO, Gene Ontology; IVs, Instrumental Variables; IVW, Inverse Variance Weighted; KEGG, Kyoto Encyclopedia of Genes and Genomes; LD, Linkage disequilibrium; LECs, Lens epithelial cells; MR, Mendelian Randomization; MGI, Mouse Genome Informatics; OD, Ocular disease; OR, Odds ratio; PPI, Protein–protein interaction; pQTL, protein Quantitative Trait Loci; PWAS, Proteome-Wide Association Study; RGCs, Retinal anglion cells; ROS/MAP, Religious Orders Study/Memory and Aging Project; SNP, Single nucleotide polymorphisms.
